# Supplementary material for: Reference Data Set for Circular Dichroism Spectroscopy Comprised of Validated Intrinsically Disordered Protein Models
Source: Appl Spectrosc. 2024 Apr 22;78(9):897–911. doi: 10.1177/00037028241239977 (PMC11453034; doi:10.1177/00037028241239977)
Supplement: sj-docx-1-asp-10.1177_00037028241239977 - Supplemental material for Reference Data Set for Circular Dichroism Spectroscopy Comprised of Validated Intrinsically Disordered Protein Models [file sj-docx-1-asp-10.1177_00037028241239977.docx]

Supplemental Material

**Reference Data Set for Circular Dichroism Spectroscopy Comprised of Validated Intrinsically Disordered Protein Models**

Gabor Nagy^1^*, Søren Vrønning Hoffman^2^, Nykola C. Jones,^2^ and Helmut Grubmüller^1^

^1^Department of Theoretical and Computational Biophysics, Max Planck Institute for Multidisciplinary Sciences, Göttingen, Germany

^2^ISA, Department of Physics and Astronomy, Aarhus University, Aarhus, Denmark

*Corresponding author: gabor.nagy@mpinat.mpg.de

Keywords: Intrinsically disordered proteins, circular dichroism spectroscopy, CD, reference data set, CD prediction, secondary structure estimation, protein ensemble refinement

**Figures**


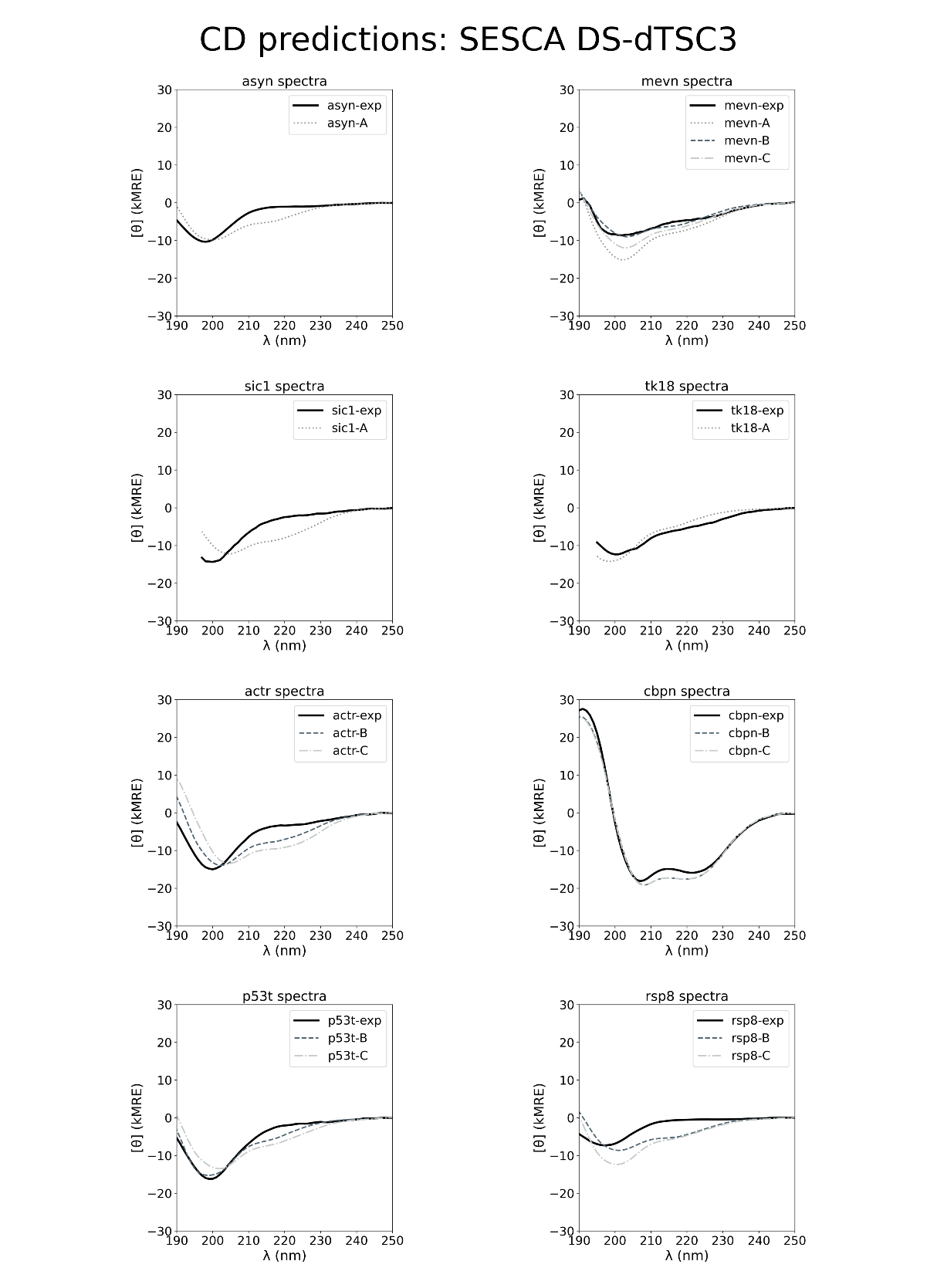


**Figure S1.** Comparison between measured CD spectra (exp), and CD spectra predicted from IDP8 ensemble models of groups A, B, and C, using the SESCA basis set DS-dTSC3.


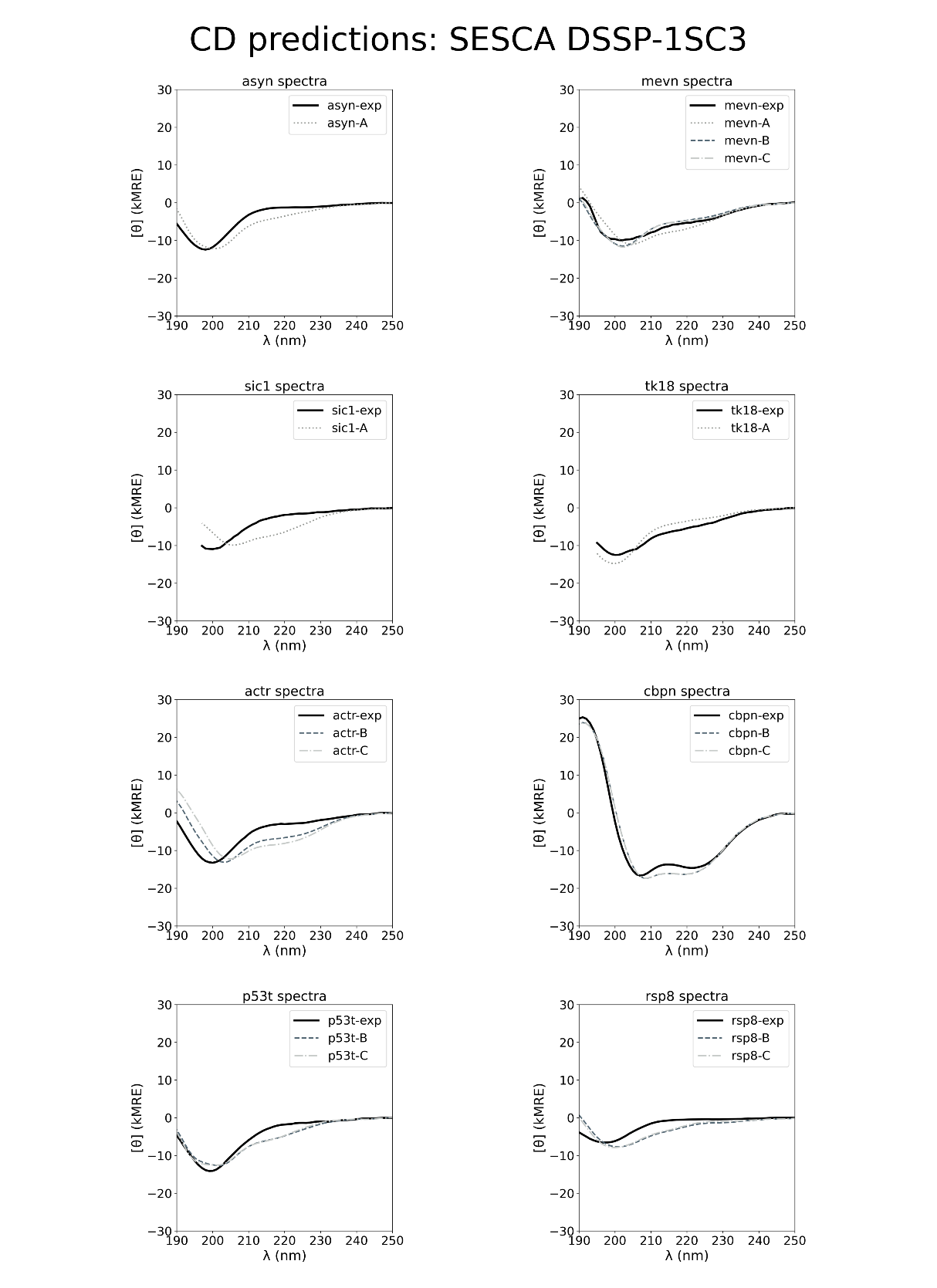


**Figure S2.** Comparison between measured CD spectra (exp), and CD spectra predicted from IDP8 ensemble models of groups A, B, and C, using the SESCA basis set DSSP-1SC3.


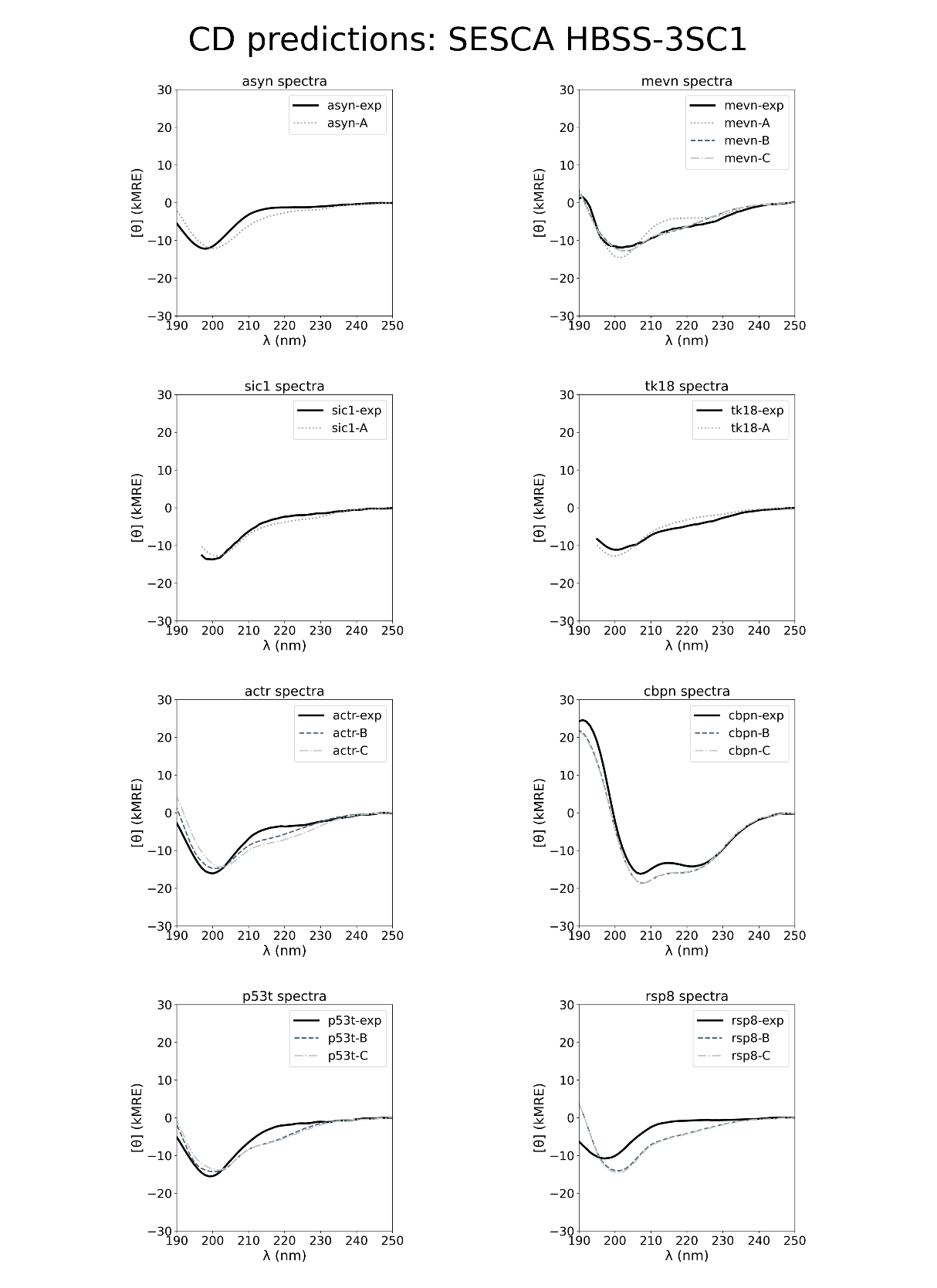


**Figure S3.** Comparison between measured CD spectra (exp), and CD spectra predicted from IDP8 ensemble models of groups A, B, and C, using the SESCA basis set HBSS-3SC1.


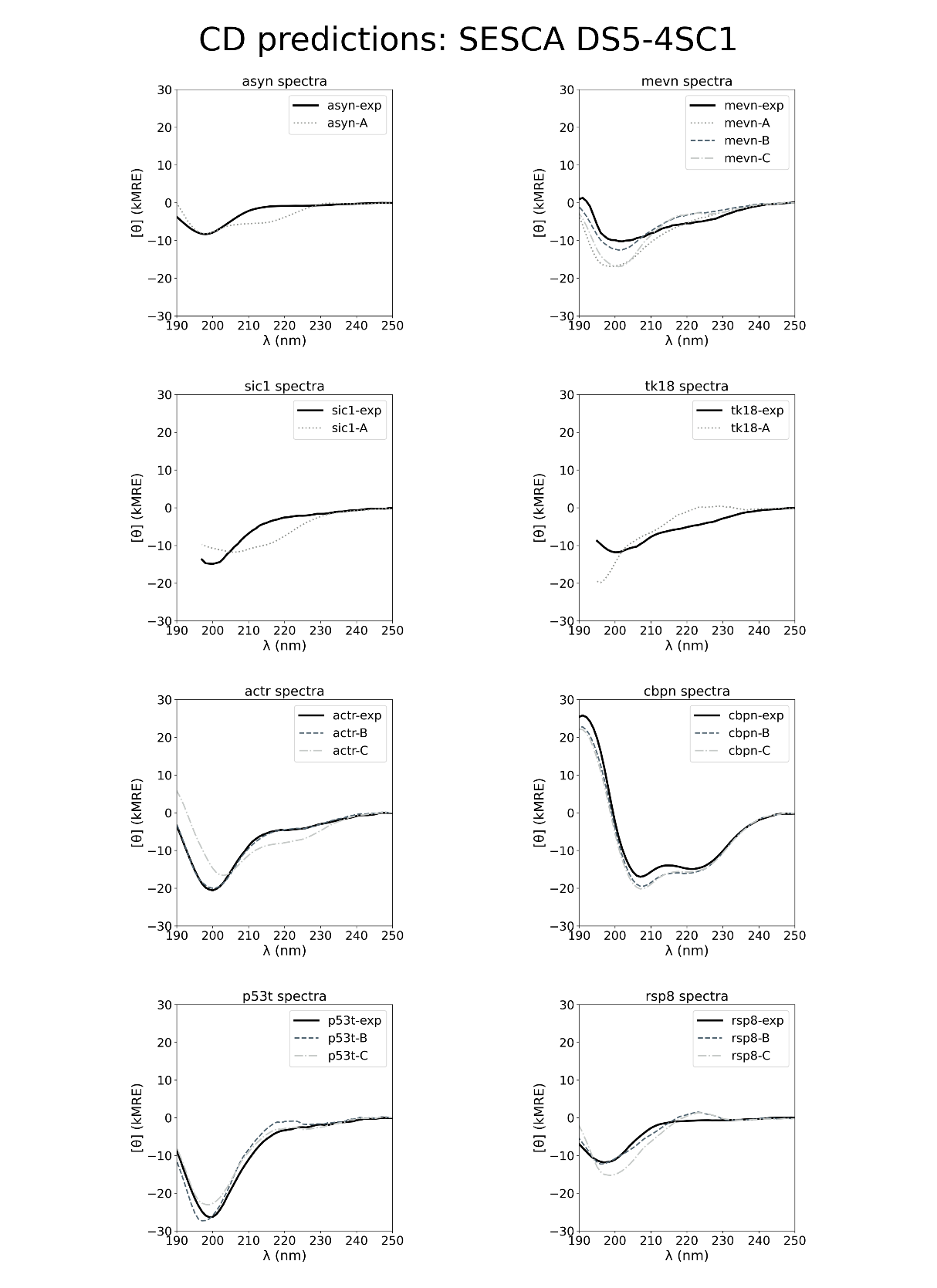


**Figure S4.** Comparison between measured CD spectra (exp), and CD spectra predicted from IDP8 ensemble models of groups A, B, and C, using the SESCA basis set DS5-4SC1.


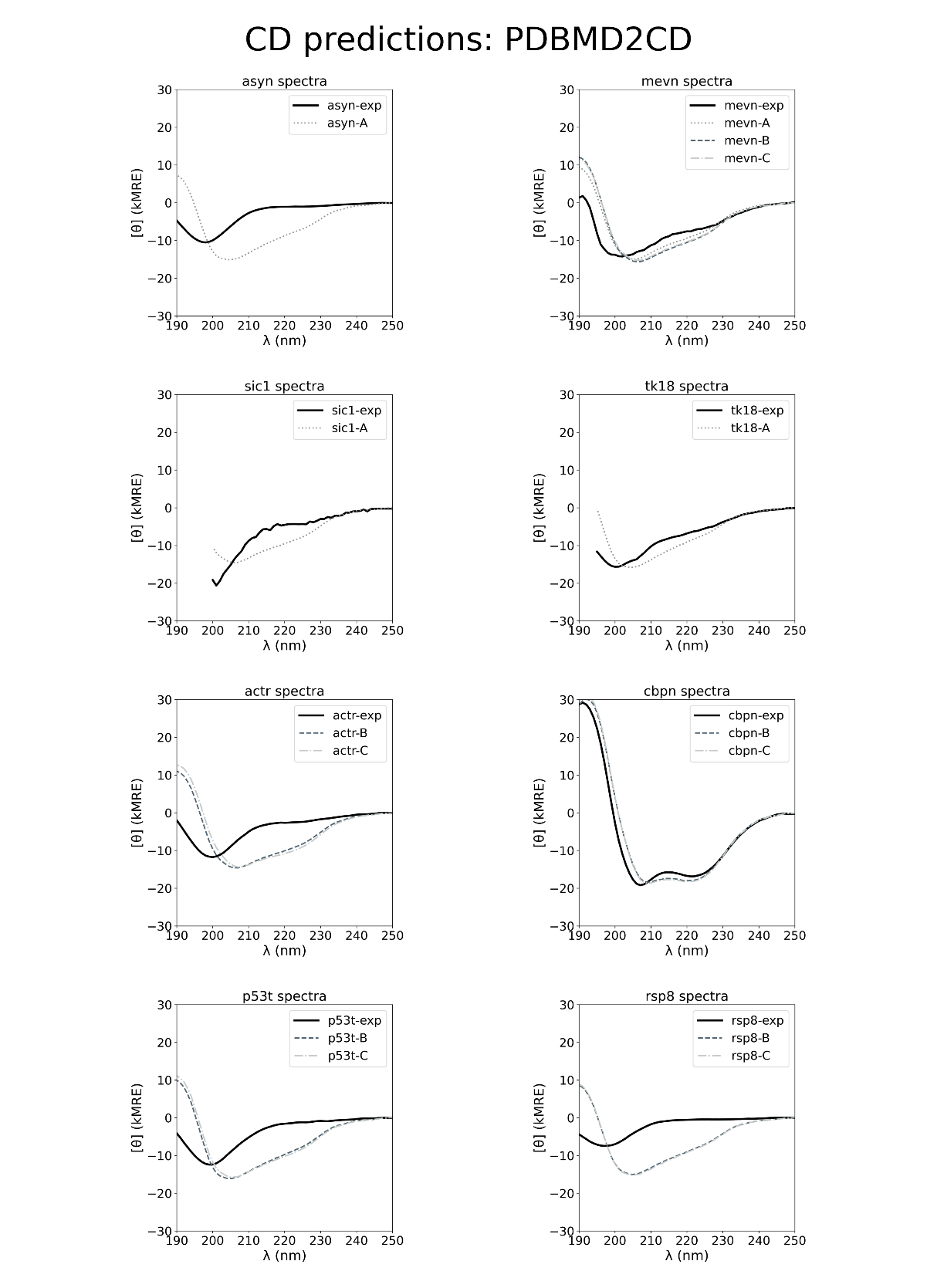


**Figure S5.** Comparison between measured CD spectra (exp), and CD spectra predicted from IDP8 ensemble models of groups A, B, and C, using the PDBMD2CD prediction server.


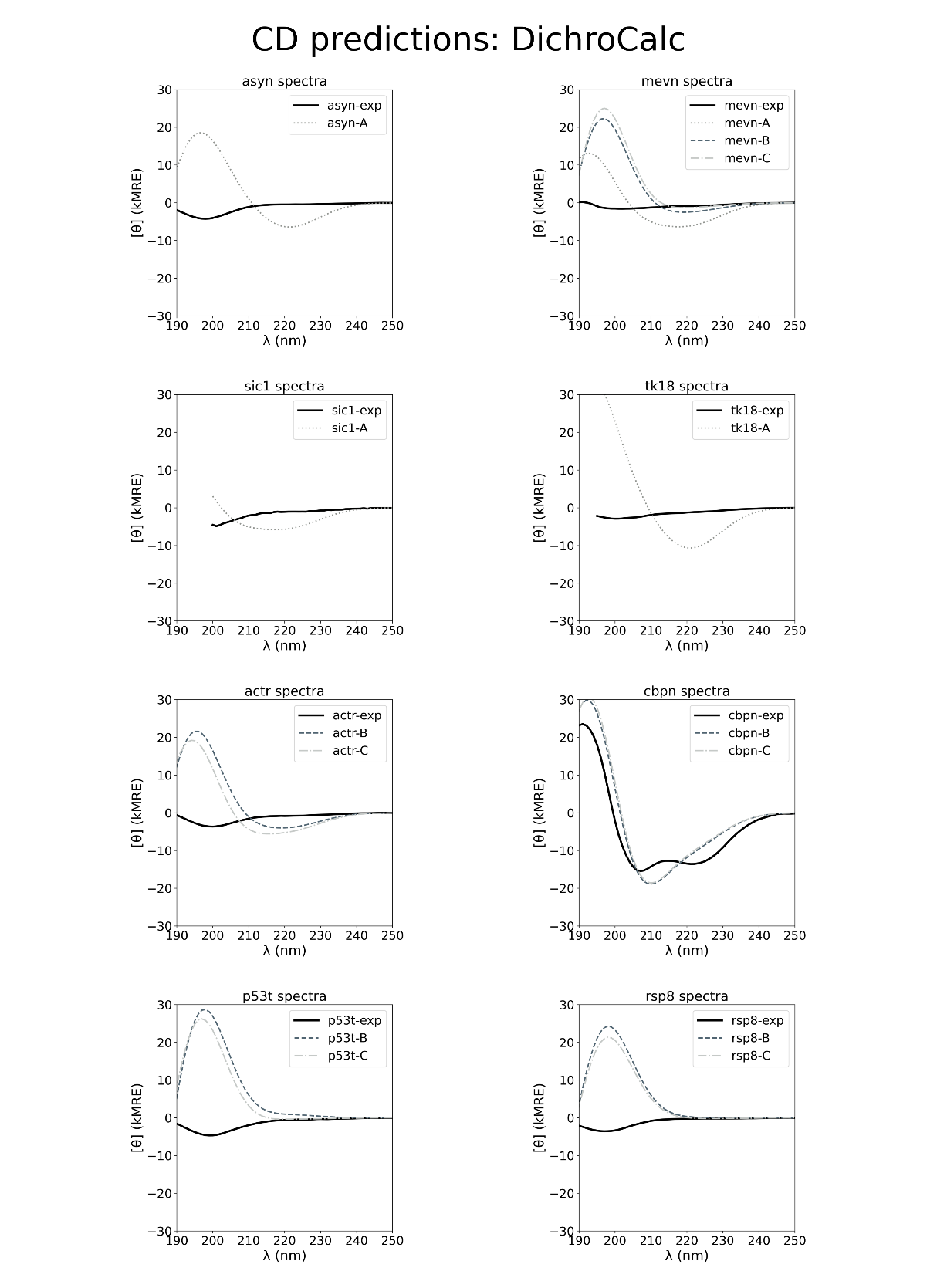


**Figure S6.** Comparison between measured CD spectra (exp), and CD spectra predicted from IDP8 ensemble models of groups A, B, and C, using the DichroCalc algorithm.

# **Tables**

**Table S1.** Bioinformatics resources. A list of online tools used in this work, including the name of the tool (resource), its main function , relevant citation (ref) used in the main publication, and URL link to access the tool.

| Resource | Function | Ref. | URL |
| --- | --- | --- | --- |
| SESCA | CD prediction | 11 | https://www.mpinat.mpg.de/sesca |
| DichroCalc | CD prediction | 5 | https://comp.chem.nottingham.ac.uk/dichrocalc |
| PDB2CD | CD prediction | 8 | https://pdb2cd.cryst.bbk.ac.uk |
| SESCA_bayes | SS estimation | 75 | https://www.mpinat.mpg.de/sesca |
| K2D3 | SS estimation | 6 | http://cbdm-01.zdv.uni-mainz.de/~andrade/k2d3 |
| BESTSEL | SS estimation | 7 | https://bestsel.elte.hu/index.php |
| SELCON3 | SS estimation | 9 | https://sites.google.com/view/sreerama |
| CCA | SS estimation | 10 | https://www.chem.elte.hu/departments/jimre |
| BRMB | database | 41 | https://bmrb.io |
| Dichroweb | database | 2 | http://dichroweb.cryst.bbk.ac.uk/html/home.shtml |
| PCDDB | database | 12 | https://pcddb.cryst.bbk.ac.uk |
| PED | database | 40 | https://proteinensemble.org |

**Table S2.** MD simulations and parameters. Summary of all MD simulations used for ensemble refinement of the newly derived IDP8 models. Different columns indicate simulation parameters for different IDP domains (abbreviations shown in the first row). The subsequent rows indicate simulation parameters used for all trajectories, temperature (T) in degrees Celsius, pressure (P) in atmospheres, and ion concentration in mol/dm^3^ (C_ion_). The rows further below describe MD trajectories, separated by horizontal lines, indicating the used force field (FF), the number of calculated trajectories (Ntraj), total simulation time (tsim), and the number of frames used for ensemble refinement (Nfr). Force fields are abbreviated as A99SB-disp (Amber99SB with dispersion corrections),^1^ A99SB-ws (Amber99SB with rescaled TIP4P water),^2^ A03-ws (Amber03 with rescaled water interactions),^3^ A14SB-OPC (Amber14SB^4^ with an OPC water model^5^), C22S-TIPS3P (CHARMM22 star with modified TIP3P water),^6^ and C36M-OPC (CHARMM36M with OPC water model).^7^

| System | mevn | actr | cbpn | p53t | rsp8 |
| --- | --- | --- | --- | --- | --- |
| T (C°) | 25 | 25 | 25 | 25 | 25 |
| P (atm) | 1 | 1 | 1 | 1 | 1 |
| Cion (M) | 150 | 50 | 50 | 150 | 50 |
| FF | A99SB-disp | A03-ws | A03-ws | A99SB-ws | A03-ws |
| Ntraj | 3 | 2 | 2 | 30 | 1 |
| tsim (us) | 30 | 9 | 20 | 600 | 10 |
| Nfr | 12 000 | 2400 | 5000 | 12000 | 2000 |
| FF | C36M-OPC | C22S-TIPS3P | A99SB-disp | C36M-OPC | C36M-OPC |
| Ntraj | 6 | 3 | 20 | 20 | 5 |
| tsim (us) | 42 | 30 | 100 | 200 | 25 |
| Nfr | 33000 | 7500 | 20000 | 20000 | 2500 |
| FF |  |  | C36M-OPC |  | C22S-TIPS3P |
| Ntraj |  |  | 20 |  | 1 |
| tsim (us) |  |  | 100 |  | 1 |
| Nfr |  |  | 10000 |  | 1000 |
| FF |  |  | A14SB-OPC |  |  |
| Ntraj |  |  | 20 |  |  |
| tsim (us) |  |  | 100 |  |  |
| Nfr |  |  | 10000 |  |  |

**Table S3.** Bayesian Maximum Entropy refinement parameters. The table summarizes details of Bayesian maximum entropy (BME) refinement of IDP8 ensemble models. Columns denote different disordered models (abbreviations of the model shown in the first row). The subsequent rows show initial ensemble size (N0), Theta scaling parameter (Θ) and final ensemble size (Nf) for group B and group C refinements. Both sets of refinements started from the same respective initial ensemble (described in Methods) using different experimental data.

| System | mevn | actr | cbpn | p53t | rsp8 |
| --- | --- | --- | --- | --- | --- |
| N0 | 45 000 | 10000 | 45000 | 32000 | 5500 |
| Θ-B | 10 | 5 | 5 | 5 | 20 |
| Nf-B | 5x20 | 5x20 | 5x20 | 1X50 | 5x50 |
| Θ-C | 20 | 10 | 10 | 5 | 10 |
| Nf-C | 5X20 | 5X20 | 5x20 | 5x50 | 5x50 |

**Table S4.** Comparison between reference SS fractions and SS fractions estimated from measured CD spectra using the SESCA basis DS-dTSC3. Solid horizontal lines separate the three ensemble model groups (A, B, and C) the reference structure belongs to. Comparisons used to compute average SS estimation accuracy are shown in bold.

| DS-dTSC3 | Ref | Est | Ref | Est | Ref | Est |
| --- | --- | --- | --- | --- | --- | --- |
| ref. model | Alpha | Alpha | Beta | Beta | Coil | Coil |
| **asyn-A** | **0.02** | **0.00 (0.03)** | **0.13** | **0.03 (0.08)** | **0.85** | **0.97 (0.09)** |
| mevn-A | 0.08 | 0.03 (0.04) | 0.02 | 0.14 (0.06) | 0.90 | 0.83 (0.07) |
| **sic1-A** | **0.12** | **0.01 (0.03)** | **0.05** | **0.03 (0.07)** | **0.83** | **0.96 (0.09)** |
| **tk18-A** | **0.01** | **0.03 (0.05)** | **0.03** | **0.02 (0.07)** | **0.97** | **0.95 (0.09)** |
| **mevn-B** | **0.06** | **0.03 (0.04)** | **0.13** | **0.14 (0.06)** | **0.80** | **0.83 (0.07)** |
| **actr-B** | **0.08** | **0.01 (0.04)** | **0.05** | **0.01 (0.06)** | **0.87** | **0.98 (0.09)** |
| **cbpn-B** | **0.41** | **0.36 (0.05)** | **0.03** | **0.03 (0.05)** | **0.56** | **0.62 (0.05)** |
| **p53t-B** | **0.00** | **0.01 (0.04)** | **0.06** | **0.02 (0.07)** | **0.94** | **0.98 (0.09)** |
| **rsp8-B** | **0.03** | **0.01 (0.04)** | **0.17** | **0.06 (0.06)** | **0.80** | **0.94 (0.08)** |
| mevn-C | 0.07 | 0.03 (0.04) | 0.05 | 0.14 (0.06) | 0.87 | 0.83 (0.07) |
| actr-C | 0.15 | 0.01 (0.04) | 0.06 | 0.01 (0.06) | 0.79 | 0.98 (0.09) |
| cbpn-C | 0.41 | 0.36 (0.05) | 0.02 | 0.03 (0.05) | 0.57 | 0.62 (0.05) |
| p535t-C | 0.05 | 0.01 (0.04) | 0.06 | 0.02 (0.07) | 0.89 | 0.98 (0.09) |
| rsp8-C | 0.03 | 0.01 (0.04) | 0.08 | 0.06 (0.06) | 0.90 | 0.94 (0.08) |

**Table S5.** Comparison between reference SS fractions and SS fractions estimated from measured CD spectra using the SESCA basis DSSP-1SC3. Solid horizontal lines separate the three ensemble model groups (A, B, and C) the reference structure belongs to. Comparisons used to compute average SS estimation accuracy are shown in bold.

| DSSP-1SC3 | Ref | Est | Ref | Est | Ref | Est | Ref | Est |
| --- | --- | --- | --- | --- | --- | --- | --- | --- |
| ref. model | Helix | Helix | Strand | Strand | Bridge | Bridge | Other | Other |
| **asyn-A** | **0.01** | **0.01 (0.04)** | **0.01** | **0.13 (0.11)** | **0.01** | **0.12 (0.04)** | **0.96** | **0.74 (0.16)** |
| mevn-A | 0.11 | 0.07 (0.05) | 0.00 | 0.28 (0.11) | 0.00 | 0.07 (0.06) | 0.89 | 0.58 (0.20) |
| **sic1-A** | **0.10** | **0.06 (0.06)** | **0.00** | **0.15 (0.12)** | **0.00** | **0.09 (0.05)** | **0.89** | **0.70 (0.20)** |
| **tk18-A** | **0.00** | **0.09 (0.07)** | **0.02** | **0.09 (0.11)** | **0.02** | **0.07 (0.05)** | **0.95** | **0.76 (0.20)** |
| **mevn-B** | **0.07** | **0.07 (0.05)** | **0.02** | **0.28 (0.11)** | **0.02** | **0.07 (0.06)** | **0.90** | **0.58 (0.20)** |
| **actr-B** | **0.11** | **0.06 (0.05)** | **0.03** | **0.09 (0.10)** | **0.02** | **0.10 (0.05)** | **0.84** | **0.75 (0.17)** |
| **cbpn-B** | **0.43** | **0.46 (0.08)** | **0.00** | **0.12 (0.10)** | **0.00** | **0.08 (0.05)** | **0.57** | **0.34 (0.16)** |
| **p53t-B** | **0.02** | **0.02 (0.04)** | **0.01** | **0.08 (0.11)** | **0.01** | **0.11 (0.04)** | **0.96** | **0.79 (0.17)** |
| **rsp8-B** | **0.04** | **0.01 (0.03)** | **0.04** | **0.20 (0.11)** | **0.01** | **0.12 (0.04)** | **0.91** | **0.68 (0.15)** |
| mevn-C | 0.08 | 0.07 (0.05) | 0.02 | 0.28 (0.11) | 0.03 | 0.07 (0.06) | 0.88 | 0.58 (0.20) |
| actr-C | 0.15 | 0.06 (0.05) | 0.05 | 0.09 (0.10) | 0.01 | 0.10 (0.05) | 0.79 | 0.75 (0.17) |
| cbpn-C | 0.43 | 0.46 (0.08) | 0.00 | 0.12 (0.10) | 0.00 | 0.08 (0.05) | 0.57 | 0.34 (0.16) |
| p535t-C | 0.00 | 0.02 (0.04) | 0.00 | 0.08 (0.11) | 0.00 | 0.11 (0.04) | 1.00 | 0.79 (0.17) |
| rsp8-C | 0.03 | 0.01 (0.03) | 0.04 | 0.20 (0.11) | 0.01 | 0.12 (0.04) | 0.92 | 0.68 (0.15) |

**Table S6.** Comparison between reference SS fractions and SS fractions estimated from measured CD spectra using the SESCA basis HBSS-3SC1. Solid horizontal lines separate the three ensemble model groups (A, B, and C) the reference structure belongs to. Comparisons used to compute average SS estimation accuracy are shown in bold.

| HBSS-3SC1 | Ref | Est | Ref | Est | Ref | Est | Ref | Est | Ref | Est |
| --- | --- | --- | --- | --- | --- | --- | --- | --- | --- | --- |
| ref. model | Helix_reg | Helix_reg | Helix_irr | Helix_irr | Beta_all | Beta_all | Turn | Turn | Other | Other |
| **asyn-A** | **0.00** | **0.00 (0.03)** | **0.00** | **0.02 (0.04)** | **0.00** | **0.02 (0.06)** | **0.49** | **0.46 (0.29)** | **0.50** | **0.50 (0.30)** |
| mevn-A | 0.02 | 0.01 (0.03) | 0.05 | 0.08 (0.08) | 0.00 | 0.07 (0.07) | 0.57 | 0.37 (0.24) | 0.36 | 0.46 (0.22) |
| **sic1-A** | **0.02** | **0.01 (0.03)** | **0.02** | **0.04 (0.06)** | **0.00** | **0.02 (0.05)** | **0.47** | **0.33 (0.22)** | **0.49** | **0.60 (0.24)** |
| **tk18-A** | **0.00** | **0.02 (0.03)** | **0.00** | **0.05 (0.06)** | **0.01** | **0.02 (0.04)** | **0.39** | **0.21 (0.22)** | **0.60** | **0.71 (0.24)** |
| **mevn-B** | **0.06** | **0.01 (0.03)** | **0.01** | **0.08 (0.08)** | **0.02** | **0.07 (0.07)** | **0.09** | **0.37 (0.24)** | **0.83** | **0.46 (0.22)** |
| **actr-B** | **0.02** | **0.01 (0.02)** | **0.01** | **0.03 (0.05)** | **0.03** | **0.02 (0.05)** | **0.18** | **0.30 (0.20)** | **0.75** | **0.65 (0.21)** |
| **cbpn-B** | **0.35** | **0.33 (0.06)** | **0.02** | **0.09 (0.08)** | **0.00** | **0.02 (0.04)** | **0.16** | **0.27 (0.18)** | **0.46** | **0.29 (0.16)** |
| **p53t-B** | **0.00** | **0.00 (0.02)** | **0.01** | **0.02 (0.05)** | **0.01** | **0.02 (0.04)** | **0.08** | **0.45 (0.28)** | **0.91** | **0.51 (0.28)** |
| **rsp8-B** | **0.01** | **0.00 (0.02)** | **0.01** | **0.01 (0.04)** | **0.03** | **0.02 (0.05)** | **0.18** | **0.44 (0.25)** | **0.78** | **0.52 (0.26)** |
| mevn-C | 0.05 | 0.01 (0.03) | 0.01 | 0.08 (0.08) | 0.02 | 0.07 (0.07) | 0.10 | 0.37 (0.24) | 0.82 | 0.46 (0.22) |
| actr-C | 0.07 | 0.01 (0.02) | 0.02 | 0.03 (0.05) | 0.02 | 0.02 (0.05) | 0.18 | 0.30 (0.20) | 0.71 | 0.65 (0.21) |
| cbpn-C | 0.35 | 0.33 (0.06) | 0.02 | 0.09 (0.08) | 0.00 | 0.02 (0.04) | 0.16 | 0.27 (0.18) | 0.48 | 0.29 (0.16) |
| p535t-C | 0.01 | 0.00 (0.02) | 0.02 | 0.02 (0.05) | 0.01 | 0.02 (0.04) | 0.11 | 0.45 (0.28) | 0.85 | 0.51 (0.28) |
| rsp8-C | 0.01 | 0.00 (0.02) | 0.01 | 0.01 (0.04) | 0.01 | 0.02 (0.05) | 0.18 | 0.44 (0.25) | 0.79 | 0.52 (0.26) |

**Table S7.** Comparison between reference SS fractions and SS fractions estimated from measured CD spectra using the SESCA basis DS5-4SC1. Solid horizontal lines separate the three ensemble model groups (A, B, and C) the reference structure belongs to. Comparisons used to compute average SS estimation accuracy are shown in bold.

| DS5-4SC1 | Ref | Est | Ref | Est | Ref | Est | Ref | Est | Ref | Est | Ref | Est |
| --- | --- | --- | --- | --- | --- | --- | --- | --- | --- | --- | --- | --- |
| ref. model | Helix1 | Helix1 | Helix2 | Helix2 | Beta1 | Beta1 | Turn1 | Turn1 | Turn2 | Turn2 | Other | Other |
| **asyn-A** | **0.02** | **0.02 (0.03)** | **0.01** | **0.04 (0.05)** | **0.13** | **0.05 (0.06)** | **0.11** | **0.38 (0.08)** | **0.19** | **0.30 (0.11)** | **0.55** | **0.20 (0.15)** |
| mevn-A | 0.05 | 0.04 (0.05) | 0.06 | 0.07 (0.07) | 0.02 | 0.13 (0.07) | 0.25 | 0.20 (0.09) | 0.19 | 0.29 (0.12) | 0.43 | 0.27 (0.18) |
| **sic1-A** | **0.08** | **0.04 (0.04)** | **0.05** | **0.06 (0.06)** | **0.05** | **0.07 (0.07)** | **0.13** | **0.35 (0.17)** | **0.16** | **0.26 (0.18)** | **0.54** | **0.22 (0.18)** |
| **tk18-A** | **0.01** | **0.09 (0.06)** | **0.00** | **0.07 (0.07)** | **0.03** | **0.05 (0.06)** | **0.31** | **0.36 (0.14)** | **0.09** | **0.19 (0.13)** | **0.57** | **0.24 (0.19)** |
| **mevn-B** | **0.05** | **0.04 (0.05)** | **0.02** | **0.07 (0.07)** | **0.13** | **0.13 (0.07)** | **0.23** | **0.20 (0.09)** | **0.29** | **0.29 (0.12)** | **0.27** | **0.27 (0.18)** |
| **actr-B** | **0.05** | **0.07 (0.05)** | **0.05** | **0.03 (0.04)** | **0.05** | **0.03 (0.05)** | **0.27** | **0.31 (0.10)** | **0.29** | **0.30 (0.11)** | **0.29** | **0.26 (0.16)** |
| **cbpn-B** | **0.36** | **0.35 (0.07)** | **0.06** | **0.03 (0.04)** | **0.03** | **0.04 (0.05)** | **0.15** | **0.13 (0.08)** | **0.20** | **0.25 (0.10)** | **0.19** | **0.20 (0.14)** |
| **p53t-B** | **0.00** | **0.05 (0.04)** | **0.02** | **0.02 (0.04)** | **0.06** | **0.02 (0.04)** | **0.39** | **0.37 (0.08)** | **0.32** | **0.31 (0.11)** | **0.21** | **0.22 (0.14)** |
| **rsp8-B** | **0.01** | **0.02 (0.03)** | **0.08** | **0.04 (0.05)** | **0.17** | **0.09 (0.07)** | **0.31** | **0.38 (0.08)** | **0.29** | **0.25 (0.10)** | **0.15** | **0.22 (0.15)** |
| mevn-C | 0.06 | 0.04 (0.05) | 0.03 | 0.07 (0.07) | 0.05 | 0.13 (0.07) | 0.29 | 0.20 (0.09) | 0.31 | 0.29 (0.12) | 0.26 | 0.27 (0.18) |
| actr-C | 0.12 | 0.07 (0.05) | 0.05 | 0.03 (0.04) | 0.06 | 0.03 (0.05) | 0.18 | 0.31 (0.10) | 0.29 | 0.30 (0.11) | 0.31 | 0.26 (0.16) |
| cbpn-C | 0.37 | 0.35 (0.07) | 0.05 | 0.03 (0.04) | 0.02 | 0.04 (0.05) | 0.18 | 0.13 (0.08) | 0.20 | 0.25 (0.10) | 0.18 | 0.20 (0.14) |
| p535t-C | 0.03 | 0.05 (0.04) | 0.04 | 0.02 (0.04) | 0.06 | 0.02 (0.04) | 0.35 | 0.37 (0.08) | 0.31 | 0.31 (0.11) | 0.22 | 0.22 (0.14) |
| rsp8-C | 0.02 | 0.02 (0.03) | 0.03 | 0.04 (0.05) | 0.08 | 0.09 (0.07) | 0.25 | 0.38 (0.08) | 0.32 | 0.25 (0.10) | 0.30 | 0.22 (0.15) |

**Table S8.** Comparison between reference SS fractions and SS fractions estimated from measured CD spectra using the K2D3 web application. Solid horizontal lines separate the three ensemble model groups (A, B, and C) the reference structure belongs to. Comparisons used to compute average SS estimation accuracy are shown in bold.

| K2D3 | Ref | Est | Ref | Est | Ref | Est |
| --- | --- | --- | --- | --- | --- | --- |
| ref. model | Alpha | Alpha | Beta | Beta | Coil | Coil |
| **asyn-A** | **0.02** | **0.04** | **0.13** | **0.32** | **0.85** | **0.65** |
| mevn-A | 0.08 | 0.03 | 0.02 | 0.28 | 0.90 | 0.68 |
| **sic1-A** | **0.12** | **0.06** | **0.05** | **0.21** | **0.83** | **0.73** |
| **tk18-A** | **0.01** | **0.07** | **0.03** | **0.22** | **0.97** | **0.71** |
| **mevn-B** | **0.06** | **0.03** | **0.13** | **0.28** | **0.80** | **0.68** |
| **actr-B** | **0.08** | **0.03** | **0.05** | **0.20** | **0.87** | **0.77** |
| **cbpn-B** | **0.41** | **0.46** | **0.03** | **0.10** | **0.56** | **0.44** |
| **p53t-B** | **0.00** | **0.03** | **0.06** | **0.21** | **0.94** | **0.76** |
| **rsp8-B** | **0.03** | **0.03** | **0.17** | **0.11** | **0.80** | **0.86** |
| mevn-C | 0.07 | 0.03 | 0.05 | 0.28 | 0.87 | 0.68 |
| actr-C | 0.15 | 0.03 | 0.06 | 0.20 | 0.79 | 0.77 |
| cbpn-C | 0.41 | 0.46 | 0.02 | 0.10 | 0.57 | 0.44 |
| p535t-C | 0.05 | 0.03 | 0.06 | 0.21 | 0.89 | 0.76 |
| rsp8-C | 0.03 | 0.03 | 0.08 | 0.11 | 0.90 | 0.86 |

**Table S9.** Comparison between reference SS fractions and SS fractions estimated from measured CD spectra using the BESTSEL web application. Solid horizontal lines separate the three ensemble model groups (A, B, and C) the reference structure belongs to. Comparisons used to compute average SS estimation accuracy are shown in bold.

| BESTSEL | Ref | Est | Ref | Est | Ref | Est | Ref | Est | Ref | Est | Ref | Est |
| --- | --- | --- | --- | --- | --- | --- | --- | --- | --- | --- | --- | --- |
| ref. model | Helix | Helix | Anti1 | Anti1 | Anti2 | Anti2 | Anti3 | Anti3 | Parallel | Parallel | Other | Other |
| **asyn-A** | **0.00** | **0.03** | **0.00** | **0.00** | **0.00** | **0.02** | **0.00** | **0.29** | **0.00** | **0.00** | **1.00** | **0.67** |
| mevn-A | 0.02 | 0.08 | 0.00 | 0.00 | 0.00 | 0.12 | 0.00 | 0.20 | 0.00 | 0.03 | 0.98 | 0.57 |
| **sic1-A** | **0.02** | **0.05** | **0.00** | **0.00** | **0.00** | **0.00** | **0.00** | **0.22** | **0.00** | **0.02** | **0.98** | **0.72** |
| **tk18-A** | **0.00** | **0.11** | **0.00** | **0.00** | **0.00** | **0.00** | **0.00** | **0.22** | **0.00** | **0.00** | **0.99** | **0.67** |
| **mevn-B** | **0.06** | **0.08** | **0.00** | **0.00** | **0.00** | **0.12** | **0.00** | **0.20** | **0.02** | **0.03** | **0.93** | **0.57** |
| **actr-B** | **0.02** | **0.08** | **0.00** | **0.00** | **0.00** | **0.00** | **0.01** | **0.20** | **0.02** | **0.00** | **0.95** | **0.72** |
| **cbpn-B** | **0.35** | **0.39** | **0.00** | **0.00** | **0.00** | **0.00** | **0.00** | **0.03** | **0.00** | **0.00** | **0.65** | **0.58** |
| **p53t-B** | **0.00** | **0.07** | **0.00** | **0.00** | **0.00** | **0.01** | **0.00** | **0.26** | **0.01** | **0.00** | **0.99** | **0.66** |
| **rsp8-B** | **0.01** | **0.04** | **0.00** | **0.00** | **0.00** | **0.03** | **0.01** | **0.24** | **0.02** | **0.00** | **0.97** | **0.70** |
| mevn-C | 0.05 | 0.08 | 0.00 | 0.00 | 0.00 | 0.12 | 0.00 | 0.20 | 0.02 | 0.03 | 0.93 | 0.57 |
| actr-C | 0.07 | 0.08 | 0.00 | 0.00 | 0.00 | 0.00 | 0.00 | 0.20 | 0.02 | 0.00 | 0.91 | 0.72 |
| cbpn-C | 0.35 | 0.39 | 0.00 | 0.00 | 0.00 | 0.00 | 0.00 | 0.03 | 0.00 | 0.00 | 0.65 | 0.58 |
| p535t-C | 0.01 | 0.07 | 0.00 | 0.00 | 0.00 | 0.01 | 0.00 | 0.26 | 0.01 | 0.00 | 0.98 | 0.66 |
| rsp8-C | 0.01 | 0.04 | 0.00 | 0.00 | 0.00 | 0.03 | 0.01 | 0.24 | 0.01 | 0.00 | 0.98 | 0.70 |

**Table S10.** Computed RMSD^SS^ values per reference model. Root-mean-squared deviations were computed from SS fractions estimated from the measured CD spectra by a given method (columns) and the SS fractions of a corresponding refence ensemble (rows). Solid horizontal lines separate the three ensemble model groups (A, B, and C). SS fractions for all SESCA basis sets (DS-dTSC3, DSSP-1SC3 HBSS-3SC1, and DS5-4SC1) as well as the SS estimation methods K2D3 and BESTSEL are shown in Tables S2-S6. The reference models used to compute the average accuracy of each method is shown in bold.

| Ref. model | DS-dTSC3 | DSSP-1SC3 | HBSS-3SC1 | DS5-4SC1 | K2D3 | BESTSEL |
| --- | --- | --- | --- | --- | --- | --- |
| **asyn-A** | **0.09** | **0.14** | **0.02** | **0.19** | **0.16** | **0.18** |
| mevn-A | 0.08 | 0.21 | 0.11 | 0.09 | 0.20 | 0.19 |
| **sic1-A** | **0.10** | **0.13** | **0.08** | **0.17** | **0.11** | **0.14** |
| **tk18-A** | **0.02** | **0.11** | **0.10** | **0.15** | **0.19** | **0.17** |
| **mevn-B** | **0.03** | **0.21** | **0.21** | **0.03** | **0.11** | **0.17** |
| **actr-B** | **0.08** | **0.07** | **0.07** | **0.02** | **0.11** | **0.13** |
| **cbpn-B** | **0.05** | **0.14** | **0.09** | **0.03** | **0.07** | **0.03** |
| **p53t-B** | **0.03** | **0.11** | **0.24** | **0.02** | **0.14** | **0.18** |
| **rsp8-B** | **0.10** | **0.15** | **0.16** | **0.06** | **0.05** | **0.15** |
| mevn-C | 0.06 | 0.20 | 0.21 | 0.05 | 0.18 | 0.17 |
| actr-C | 0.14 | 0.07 | 0.07 | 0.06 | 0.11 | 0.11 |
| cbpn-C | 0.04 | 0.14 | 0.10 | 0.04 | 0.07 | 0.04 |
| p53t-C | 0.06 | 0.13 | 0.21 | 0.02 | 0.11 | 0.17 |
| rsp8-C | 0.03 | 0.16 | 0.17 | 0.07 | 0.03 | 0.15 |

**References**

1. P. Robustelli, S. Piana, D.E. Shaw. “Developing a Molecular Dynamics Force Field for Both Folded and Disordered Protein States”. Proc. Natl. Acad. Sci. U.S.A. 2018. 115(21). 10.1073/pnas.1800690115

2. R.B. Best, W. Zheng, J. Mittal. “Balanced Protein–Water Interactions Improve Properties of Disordered Proteins and Non-Specific Protein Association”. J. Chem. Theory Comput. 2014. 10(11): 5113–5124. 10.1021/ct500569b

3. Y. Duan, C. Wu, S. Chowdhury, M.C. Lee, G. Xiong, W. Zhang, et al. “A Point-Charge Force Field for Molecular Mechanics Simulations of Proteins Based on Condensed-Phase Quantum Mechanical Calculations”. J. Comput. Chem. 2003. 24(16): 1999–2012. 10.1002/jcc.10349

4. J.A. Maier, C. Martinez, K. Kasavajhala, L. Wickstrom, et al. “Ff14SB: Improving the Accuracy of Protein Side Chain and Backbone Parameters from ff99SB”. J. Chem. Theory Comput. 2015. 11(8): 3696–3713. 10.1021/acs.jctc.5b00255

5. S. Izadi, R. Anandakrishnan, A.V. Onufriev. “Building Water Models: A Different Approach”. J. Phys. Chem. Lett. 2014. 5(21): 3863–3871. 10.1021/jz501780a

6. A.D. Mackerell Jr., D. Bashford, M. Bellott, R.L. Dunbrack Jr., et al. “All-Atom Empirical Potential for Molecular Modeling and Dynamics Studies of Proteins”. J. Phys. Chem. B. 1998. 102(18): 3586–3616. 10.1021/jp973084f

7. J. Huang, S. Rauscher, G. Nawrocki, T. Ran, M. Feig, B.L. de Groot, et al. “CHARMM36m: An Improved Force Field for Folded and Intrinsically Disordered Proteins”. Nat. Methods. 2017. 14(1): 71–73. 10.1038/nmeth.4067
